# Temporal Dynamics of Host Plant Use and Parasitism of Three Stink Bug Species: A Multi-Trophic Perspective

**DOI:** 10.3390/insects16070731

**Published:** 2025-07-17

**Authors:** Martina Falagiarda, Francesco Tortorici, Sara Bortolini, Martina Melchiori, Manfred Wolf, Luciana Tavella

**Affiliations:** 1 Institute for Plant Health, Research Centre Laimburg, Laimburg 6, 39040 Auer, Italy; sara.bortolini@laimburg.it (S.B.); martina.melchiori@laimburg.it (M.M.); manfred.wolf@laimburg.it (M.W.); 2 Department of Agricultural, Forest and Food Sciences, University of Torino, Largo Paolo Braccini 2, 10095 Grugliasco, Italy; luciana.tavella@unito.it

**Keywords:** Pentatomidae, Scelionidae, multi-trophic interactions, phenology, biological control

## Abstract

Stink bugs are widespread agricultural pests that damage crops and reduce yield. Their impact is influenced by host plant selection and interactions with natural enemies, particularly egg parasitoids. Understanding these relationships is crucial for improving biological control strategies. This paper investigates the seasonal host plant use and parasitism of *Halyomorpha halys*, *Palomena prasina*, and *Pentatoma rufipes* in South Tyrol, Italy. Over two years, we conducted field surveys at 27 sites, recording stink bug presence across 85 plant species and analyzing egg parasitism rates. Results show that stink bugs exhibit distinct host plant preferences, with *H. halys* utilizing the broadest range of host plants while *P. prasina* and *P. rufipes* showed stronger affinities for specific families such as Sapindaceae and Rosaceae. Parasitism rates varied across species and plant families: *Trissolcus japonicus* predominantly parasitized *H. halys* while *T. cultratus* and two *Telenomus* species targeted *P. rufipes* and *P. prasina*, respectively. Spatial–temporal features and host plant associations significantly influenced species distributions and parasitoid occurrence. These findings emphasize the role of plant–insect interactions in shaping pest and parasitoid dynamics. Integrating plant diversity into pest management strategies could enhance parasitoid effectiveness and reduce stink bug populations, contributing to more sustainable agricultural practices.

## Simple Summary

Stink bugs are major agricultural pests that damage crops and reduce yield. This paper examines how three stink bug species—the brown marmorated stink bug, green shield bug, and red-legged shield bug—use different host plants and how egg parasitoids interact with them. Field surveys in South Tyrol, Italy revealed that stink bugs prefer certain plant families, with maple trees being a key resource. Parasitism rates varied, with *Trissolcus japonicus* targeting *Halyomorpha halys*, *Trissolcus cultratus* attacking *Pentatoma rufipes*, and two *Telenomus* species parasitizing *Palomena prasina*. These findings highlight how plant diversity influences pest and parasitoid interactions, providing insights for sustainable pest management.

## 1. Introduction

Stink bugs (Hemiptera: Pentatomidae) represent major agricultural pests worldwide, causing extensive economic losses across a wide range of crops [1,2,3]. Their economic impact has become increasingly significant, particularly due to their polyphagy and feeding behavior, which involves piercing plant tissues and injecting digestive enzymes [2,4]. This feeding mechanism results in the severe quality degradation of agricultural products, affecting both yield and market value [2,3].

Stink bug management is often complex due to the diverse species composition and ecological patterns within agricultural landscapes. Species can display distinct temporal dynamics and host plant preferences and can overlap in their impact on crops [5]. Understanding these patterns is crucial as stink bugs use both cultivated and wild host plants throughout their life cycles. The presence of non-cultivated host plants in agroecosystems significantly influences their population dynamics and can affect the efficacy of targeted control strategies, including conservation biological control approaches [6]. Consequently, effective management requires a thorough understanding of local species assemblages and their interactions with the surrounding landscape.

Among the most common stink bug species in South Tyrol, a region of northern Italy, are *Halyomorpha halys* (Stål), *Palomena prasina* (L.), and *Pentatoma rufipes* (L.), each of which has distinct characteristics and agricultural impacts [7,8,9,10]. *Halyomorpha halys*, the brown marmorated stink bug, is an invasive agricultural pest native to East Asia that has caused considerable economic damage to crops including apples, pears, peaches, and soybean across Europe since its introduction in 2007 [11,12]. *Palomena prasina*, the green shield bug, primarily feeds on a variety of plants including fruits and deciduous shrubs [13]. This species is prevalent in many regions of Europe, where it impacts crops such as hazelnuts [8,14]. *Pentatoma rufipes*, the red-legged shield bug or forest bug, is widespread in Europe and primarily feeds on the sap of deciduous trees, including oaks and hazelnuts. This species is also known to be a pest in orchards, where it can cause economic damage to fruit crops [9].

Understanding the phenology and ecology of these three stink bug species requires detailed knowledge of their host plant associations, feeding preferences, and seasonal utilization patterns across different plant communities. Despite their similarities as sap-feeders, each species demonstrates unique characteristics and behaviors that influence their interactions with local ecosystems and agricultural systems [10]. Specificity in host selection is crucial to stink bug reproductive success as some plants are essential for egg-laying and nymph development while others may only support adult feeding [15,16,17]. For instance, according to Bergmann et al. [15], maples and legumes constitute half of the top 25 hosts of *H. halys*, and host plant use varies by life stage, with host plant diversity decreasing across developmental stages: adults utilize the broadest range of plant taxa, late nymphs are found on fewer plant species, and egg masses are restricted to the narrowest host plant range. Moreover, the seasonal dynamics of *H. halys* adults and nymphs vary greatly depending on the host plant, the growth stage of the plant, and the time of year [18]. Knowing the seasonal sequence of host plant use, along with their suitability for reproduction and nymphal development, is essential for effective stink bug management and for the implementation of conservation biological control.

Parasitism represents another crucial factor influencing stink bug population dynamics, particularly in relation to their host plant associations [19]. The accurate estimation of parasitism rates is critical to evaluate the efficacy of biological control agents, especially against invasive species such as *H. halys* [20,21,22]. In South Tyrol, the release of *Trissolcus japonicus* (Ashmead) (Hymenoptera: Scelionidae) began in 2020 as part of a nationwide classical biocontrol program against *H. halys* [23]. The efficacy of these egg parasitoids is strongly influenced by host plant characteristics as plants mediate the tritrophic interactions between stink bugs and their natural enemies [24]. Plant volatile signals play a fundamental role in these interactions, with oviposition-induced plant volatiles serving as chemical cues for egg parasitoids [25,26].

For instance, when *Nezara viridula* (L.) lays eggs on plants such as the fava bean (*Vicia faba* L.), the plants emit specific volatile compounds that attract parasitoids such as *Trissolcus basalis* (Wollaston) [27]. The sophistication of this chemical communication is evident in the way the mating status of female stink bugs can influence these volatile emissions, creating a web of chemical signals that guide egg parasitoids to fresh egg masses [25,28]. Moreover, the success of parasitoids in controlling stink bug populations is closely related to the availability of plant-provided resources, particularly nectar [29,30]. According to Lee et al. [31], plants such as buckwheat (*Fagopyrum esculentum* Moench) and Indian blanket (*Gaillardia pulchella* Foug.) act as important nectar sources, enhancing parasitoid longevity and reproductive capacity. This relationship has important implications for agricultural management as the strategic incorporation of nectar-producing plants near crops can significantly increase parasitism rates and improve biological control outcomes [29,32].

Habitat diversity also plays a crucial role in shaping egg parasitoid–stink bug interactions [33,34]. Different parasitoid species show distinct habitat preferences, resulting in a diverse range of biological control agents across the landscape [20,33]. For instance, while generalist species such as *Anastatus bifasciatus* (Geoffroy) (Hymenoptera: Eupelmidae) tend to dominate in urban and forest environments, others such as *Trissolcus* spp. and *Telenomus* spp. (Hymenoptera: Scelionidae) are more prevalent in agricultural settings [10].

Given this complex ecological context, our study investigated the host plant utilization patterns of three stink bug species (*H. halys*, *P. prasina*, and *P. rufipes*) at different life stages in South Tyrol. Through two years of research, we examined three key questions: (1) How do these three stink bug species divide host plant resources across different plant families? (2) What drives their stage-specific distribution patterns on host plants? (3) How do parasitoid communities respond to these host–plant associations? By understanding these relationships, we aim to contribute to the development of more effective biological control strategies that exploit natural enemy–plant interactions, with practical implications for integrated pest management programs.

## 2. Materials and Methods

### 2.1. Study Area and Surveyed Sites

The study was conducted during 2022–2023 at 27 sites located in South Tyrol, northeastern Italy (Supplementary Table S1), covering a range of habitats and altitudinal gradients. South Tyrolean landscape is predominantly mountainous with extensive forests. Most of the cultivated area is devoted to apple production (ca. 18,400 ha), spanning the main valleys from 200 to 1100 m a.s.l. Sites were selected to ensure representative sampling and cover the major habitat types across the region, including agricultural, urban, and semi-natural environments. The surveyed sites included nine apple orchards, all managed according to integrated pest management guidelines [35], with the exception of site 19, which was an organically managed apple orchard. Nine survey sites were located in urbanized areas and were characterized by exclusively ornamental woody vegetation, representing areas with no natural vegetation patches or managed lawns. The remaining sites were located at woodland edges.

### 2.2. Field Sampling of Stink Bugs

Surveys were carried out monthly from April to September in both years to detect seasonal fluctuations in populations of the three most abundant stink bug species, namely *H. halys*, *P. prasina,* and *P. rufipes*. All surveys were conducted within the first 10 days of each month to ensure temporal standardization across sites and minimize phenological variation within sampling periods. Two complementary sampling methods, i.e., beat sheet sampling and direct visual inspection of host plants, were used at each site within a 50 m radius area. To minimize the impact of high temperatures on insect activity and detection efficiency, all surveys were conducted between 8 and 11 a.m. These two methods were employed as complementary techniques to account for different detection efficiency in relation to plant architectures and stink bug behavioral states, ensuring comprehensive sampling regardless of vegetation structure or insect activity patterns.

Monitoring covered a wide range of plant species, including shrubs and trees, with species composition varying between and within sites throughout the season. Beat sheet sampling was standardized on six different plant species at each survey site, with 15 beats per species, resulting in a total of 90 beats per survey and site. In the apple orchards, 15 beats per apple tree were performed on three apple trees per survey and site (totally 45 beats), selected on alternate rows during successive sampling. To facilitate insect collection, a plastic bag was attached to the center of the beat sheet, where a hole allowed samples to funnel into the bag for efficient retrieval. All collected stink bugs were placed in plastic bags, which were labeled and then transported to the laboratory, where they were stored in a freezer at −30 °C. Visual inspection was conducted for one person-hour for each survey and site. Stink bug species and developmental stages found during sampling were recorded directly at the site. In the laboratory, the identification of field-collected bugs was confirmed using taxonomic keys from Ribes and Pagola-Carte [36].

### 2.3. Field Collection of Eggs and Identification of Emerged Parasitoids

Egg masses were collected by direct visual inspection during each survey. Once detected, they were transferred to plastic Petri dishes (Ø 90 mm), labeled, and transported to the laboratory, where they were maintained under controlled conditions in a climatic chamber (25 ± 1 °C, 60 ± 5% RH, 16:8 h L:D) to allow for the emergence of bug nymphs or parasitoid adults. All egg masses were examined under a stereomicroscope to confirm their identification to species level following Ribes and Pagola-Carte [36]. Three to four weeks after collection, eggs were classified into four categories: hatched, unhatched, predated, or parasitized.

In some cases, parasitoids had already emerged in the field before egg collection, and consequently, they were identified based on the shape of the exit hole left on the egg chorion and the structure of the meconium, following the criteria described by Sabbatini Peverieri et al. [37]. Since *Telenomus* spp. and *Trissolcus* spp. exhibit indistinguishable exit hole morphology and meconium characteristics, we assigned these individuals to the family Scelionidae when species-level identification was not possible. This classification ensured consistency in data interpretation while acknowledging limitations in taxonomic resolution due to natural emergence prior to laboratory processing.

Parasitoids that emerged from the eggs in the laboratory were preserved in 1.5 mL Eppendorf^®^ tubes (Eppendorf SE, Hamburg, Germany) containing 70% ethanol for further identification. Then, they were identified morphologically under a stereomicroscope using the following taxonomic keys: Askew and Nieves-Aldrey [38] and Peng et al. [39] for Eupelmidae; Talamas et al. [40,41], Tortorici et al. [42], and Moraglio et al. [43] for the genus *Trissolcus* Ashmead; Tortorici et al. [44] for the genus *Telenomus* Haliday; and Sabbatini Peverieri et al. [45] for the genus *Acroclisoides* Girault and Dodd (Hymenoptera: Pteromalidae). All specimens used for morphological analysis were deposited in the collection of the Laimburg Research Centre, Institute for Plant Health, Laimburg, Italy and the Dipartimento di Scienze Agrarie, Forestali e Alimentari (DISAFA), University of Torino, Torino, Italy.

### 2.4. Data Analysis

All analyses were performed using R software version 4.3.1 (R Core Team, 2024). Seasonal patterns of stink bug distribution across host plants were visualized using heatmaps, with separate visualizations for each life stage (egg, nymph, and adult) of *H. halys*, *P. prasina*, and *P. rufipes*. Data were aggregated by month and plant family, and abundance values were represented using color gradients. The plant family category ‘Others’ included all plant families with fewer than five individuals recorded.

To analyze host plant family preferences, a Chi-square test of independence was performed to examine the relationship between plant family use and bug species, excluding the ‘Others’ category. Because of low expected frequencies in several cells, Fisher’s exact test was used to confirm the results. Standardized residuals were calculated to identify significant associations. To analyze the association between plant family preferences and species (*H. halys*, *P. prasina*, and *P. rufipes*), pairwise comparisons were conducted using both Chi-square test and Fisher’s exact test. The analysis was performed after filtering out the ‘Others’ category from the plant family variable to focus on the main plant families of interest. To account for the increased risk of Type I errors from multiple comparisons, we applied the Bonferroni correction.

Parasitism rates were calculated for each host plant family and stink bug species combination. The temporal dynamics of parasitoid emergence were analyzed using a density ridge plot, showing the number of parasitized egg masses across weeks.

To examine the relationships between parasitoid communities, spatial and temporal variables, and sampling sites, Canonical Correspondence Analysis (CCA) was applied. Only parasitized egg masses were considered for this analysis. The choice of CCA over Redundancy Analysis was based on the gradient length resulting from a preliminary Detrended Correspondence Analysis. The significance of the CCA model and individual environmental variables was tested using permutation tests (999 permutations). The CCA sorting was visualized using the ggplot2 package, with species scores, spatial and temporal vectors, and site scores displayed in a triplot. Sampling dates (from both study years) were included as supplementary points to visualize temporal changes in community composition. Variables included elevation and distance range from the closest *T. japonicus* release point (Supplementary Table S1) and week number as temporal variable.

Additionally, a host–parasitoid network analysis was conducted to explore the interactions between stink bugs and their parasitoids. This analysis involved creating a bipartite network matrix (bipartite package) representing the interactions between host species (stink bugs) and parasitoid species. Network metrics such as connectance, specialization (H2’—degree of deviation from random associations between species), and nestedness were calculated to assess the structure of these interactions.

## 3. Results

### 3.1. Seasonal Abundance

A total of 431 adults, 463 nymphs and 210 egg masses of *H. halys*, *P. prasina,* and *P. rufipes* were collected during the two-year surveys. The three stink bug species exhibited distinct temporal patterns in their life cycle stages throughout the growing season (Table 1). *Halyomorpha halys* showed limited activity during spring, with adults observed only occasionally. Population dynamics changed dramatically in mid-summer, with a peak of adult presence in August and September. Egg masses were most abundant from July to September, with the parasitism rate reaching the highest level of 44.2% in September.

*Palomena prasina* demonstrated an earlier seasonal pattern compared to *H. halys*, with reproductive activity concentrated in the early-to-mid-summer period. Egg masses were predominantly found from May to early July while adult populations showed higher values in May and August. Parasitism rates were notably high during the main reproductive period, reaching 62.9% in June and maintaining high levels through July.

*Pentatoma rufipes* displayed a unique life cycle pattern, overwintering as second-instar nymphs and maintaining nymphal populations until July. Adult emergence began in June and peaked in August, with 97 individuals recorded. The species exhibited a concentrated reproductive period in September, when most egg masses were observed. Parasitism rates were high during the reproductive period, with parasitism of 100% in August (on a single egg mass) and 52% in September (on multiple egg masses), indicating intense parasitoid pressure coinciding with oviposition activity.

### 3.2. Host Plant Utilization Patterns

**Figure 1:**
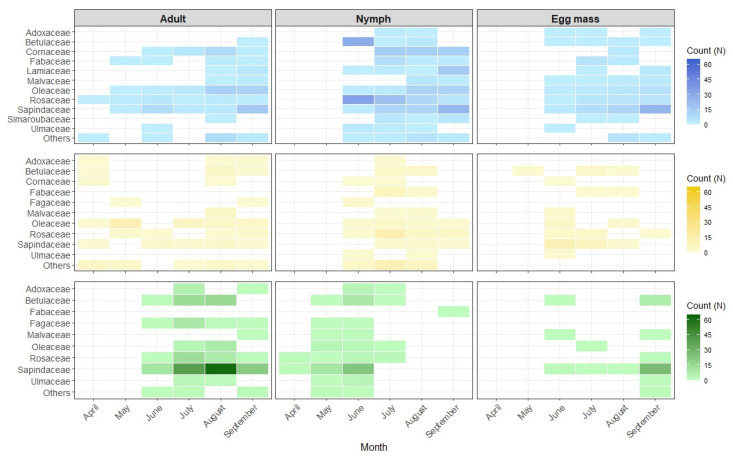
Distribution of different life stages across the plant families during the season—egg mass, nymph, and adult—for *Halyomorpha halys*, *Palomena prasina*, and *Pentatoma rufipes*.

The three stink bug species were recorded on 85 different host plants during the two-year surveys (Supplementary Table S2). The analysis of plant family preferences (Figure 1) revealed complex patterns of host plant use by the three stink bug species during the season. Sapindaceae emerged as the dominant host plant family for all three species, particularly due to the presence of *Acer* species. Both *H. halys* and *P. prasina* showed additional strong associations with Rosaceae and Oleaceae although their utilization patterns varied temporally. The preference for these plant families remained consistent in both study years, suggesting stable relationships with host plants.

Species-specific analysis revealed distinct patterns of host plant exploitation. *Halyomorpha halys* exhibited the broadest host range, utilizing several plant families but showing particular affinity for Sapindaceae and Oleaceae. Adult distribution patterns were more disperse across multiple plant families, especially on Sapindaceae (*Acer* spp.), Oleaceae (*Fraxinus* spp.), and Rosaceae (*Prunus* spp. and *Malus* spp.). Nymphal abundance showed high concentrations on Rosaceae, Betulaceae, and Sapindaceae during summer months, starting from June. Consistent oviposition activity was observed on Sapindaceae throughout the latter part of the growing season. *Palomena prasina* demonstrated generalist behavior with notable temporal variation in plant family utilization while *P. rufipes* displayed strong specificity for Sapindaceae, particularly *Acer pseudoplatanus* L. and *A. platanoides* L.

Host plant preferences were further differentiated by life stage, with adults generally showing a broader distribution across plant families compared to nymphs and eggs. Nymphal stages of all three species were found to be concentrated on specific plant families, suggesting the importance of certain host plants for immature development. Egg laying patterns revealed a strong preference for Sapindaceae across all stink bug species throughout their active period, indicating the particular importance of these plants for reproduction.

The Chi-square test showed a significant difference in the preferences of the three stink bug species in their use of plant families (χ^2^ = 55.59, df = 22, *p*-value < 0.0001; Fisher’s exact test *p* < 0.0001). The analysis of standardized residuals (Table 2) revealed several significant associations. *Halyomorpha halys* showed a significant negative association with Fagaceae (z = −2.15, *p* < 0.05) but was positively associated with both Lamiaceae (z = 2.49, *p* < 0.05) and Simaroubaceae (z = 2.23, *p* < 0.05). *Palomena prasina* did not show any significant associations with specific plant families. *Pentatoma rufipes* displayed a strong positive association with Fagaceae (z = 2.85, *p* < 0.01) and a significant negative association with Cornaceae (z = −2.06, *p* < 0.05).

Pairwise comparisons between the three stink bug species (Table 3) showed a significant difference in plant utilization between *P. rufipes* and *H. halys* (*p* < 0.001). In contrast, *P. rufipes* and *P. prasina* showed similar plant family distributions (*p* > 0.05). *Palomena prasina* and *H. halys* exhibited intermediate differences (*p* < 0.05), though this association did not remain significant after accounting for multiple comparisons.

### 3.3. Parasitism Patterns

**Figure 2:**
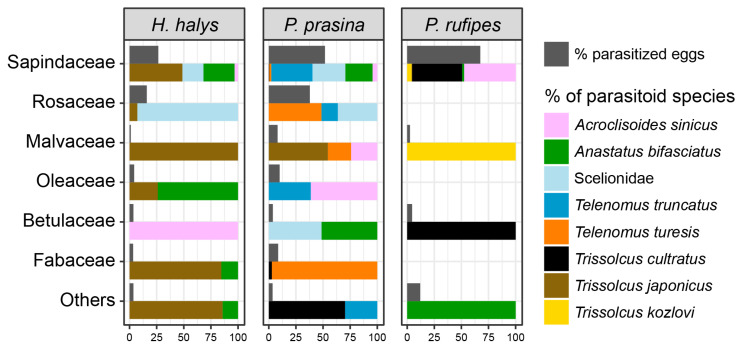
Parasitism rates for the three stink bug species (grey) and relative abundance of the parasitoid species (colored bars) across the different host plant families.

A total of 1162 parasitoids emerged from field collected egg masses, belonging to seven species. The parasitoid communities associated with each stink bug species showed distinct composition and host plant preferences (Figure 2). In *H. halys*, egg parasitism rates were the highest on Sapindaceae, where a diverse parasitoid complex was recorded, including *T. japonicus*, which was predominant, and *A. bifasciatus*. The consistent presence of these parasitoids across multiple plant families reflects their association with habitats and plant species that also harbor their hosts. However, parasitism rates on *H. halys* eggs were lower on host plants belonging to other families compared to Sapindaceae. *Palomena prasina* exhibited the most diverse parasitoid assemblage among the three species, with particularly high parasitism rates observed in both Sapindaceae and Rosaceae. The parasitoid community included *Trissolcus* spp., *A. bifasciatus*, *Telenomus truncatus* (Nees von Esenbeck), and *T. turesis* Walker. *Pentatoma rufipes* showed a more specialized parasitism pattern, with *Trissolcus cultratus* (Mayr) emerging as the primary parasitoid species and parasitism largely concentrated on Sapindaceae. Other parasitoid species occasionally emerging from *P. rufipes* eggs were *Trissolcus kozlovi* Rjachovskij and *A. bifasciatus*. The hyperparasitoid *Acroclisoides sinicus* (Huang and Liao) was recovered from eggs of all three stink bug species, with particularly frequent emergence from *P. rufipes*.

**Figure 3:**
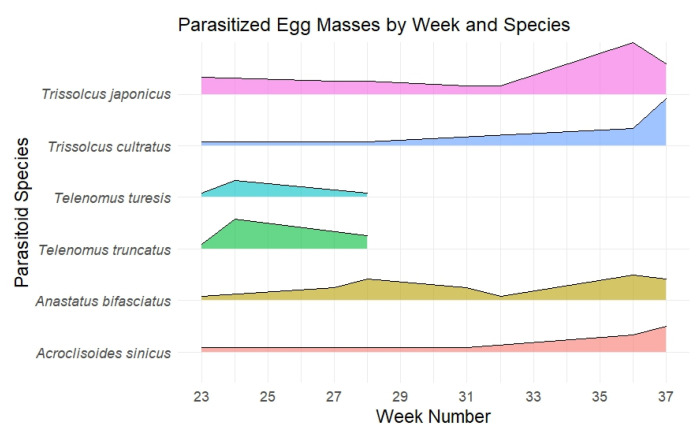
Observed phenology of parasitoid species emerged from field collected egg masses.

The analysis of parasitoid phenology (Figure 3) revealed distinct temporal patterns in parasitoid activity throughout the growing season. Two *Trissolcus* species showed extended seasonal activity: *T. japonicus* maintained presence throughout the season with increased activity in September (weeks 35–37) while *T. cultratus* demonstrated similar persistence with notable late-season abundance. This timing coincided with the peak egg-laying periods of their primary hosts. Early-season parasitism was characterized by the presence of two *Telenomus* species (*T. turesis* and *T. truncatus*), which were active from June to early July (weeks 23–27) before declining. Their activity period aligned with the reproductive phase of *P. prasina*.

The generalist parasitoid *A. bifasciatus* maintained a relatively stable presence throughout the season, showing moderate fluctuations but no dramatic peaks. The hyperparasitoid *A. sinicus* exhibited a distinct temporal pattern, gradually increasing activity as the season progressed. Its presence became more pronounced in later weeks, likely responding to the growing availability of primary parasitoids in the system.

**Figure 4:**
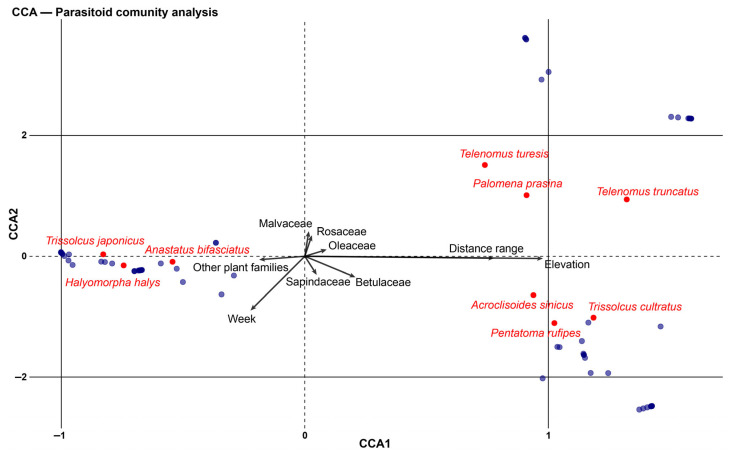
Canonical Correspondence Analysis (CCA) ordination plot showing the relationship between stink bug species and their associated parasitoids (red dots), spatial and temporal variables (black arrows), and sampling sites (blue dots).

The CCA ordination revealed the ecological patterns among stink bug species, egg parasitoid communities, and environmental variables (Figure 4). The ordination demonstrates distinct host–parasitoid relationships within the community structure. For instance, *H. halys* and *T. japonicus* showed spatial proximity in the negative region of CCA1. Similarly, *P. rufipes* showed significant correlation with *T. cultratus* while *P. prasina* demonstrated strong associations with both *Telenomus* species.

Environmental gradients emerged as primary drivers of community distribution. Elevation exhibited a strong positive correlation along CCA1, with *P. rufipes* and *P. prasina* and their associated parasitoids showing affinity for higher elevations, whereas *H. halys* demonstrated preference for lower elevations. The distance range vector, representing spatial distribution from *T. japonicus* release points, indicated the declining abundance of both *H. halys* and *T. japonicus* with increasing distance from biological control implementation sites.

Plant family associations (Malvaceae, Rosaceae, Sapindaceae, Betulaceae) demonstrated relatively low influence on species distribution patterns. The spatial heterogeneity of sampling sites, particularly evident in the positive region of CCA1, suggests habitat-specific host–parasitoid associations mediated by environmental conditions. Sampling dates (blue dots in Figure 4), pooled across both years, are positioned in the ordination space according to the parasitoid assemblages observed at each time point, illustrating seasonal shifts in species composition and host associations.

The host–parasitoid network analysis (Supplementary Figure S1) revealed a connectance of 0.62, indicating that approximately 62% of potential interactions between stink bugs and their parasitoids were realized. The specialization index (H2’) was calculated at 0.435, indicating a moderate level of specialization in species interactions, while the nestedness value of 15.27 revealed a strongly structured network, with generalist parasitoids interacting with a subset of specialized hosts.

## 4. Discussion

This paper contributes to the understanding of host plant associations and parasitoid interactions for three ecologically and agriculturally important stink bug species in the investigated region. While extensive research has documented the host plants and parasitism of *H. halys*, similar studies on *P. prasina* and *P. rufipes* remain limited.

The observed phenological patterns of the three stink bug species reveal distinct temporal niches within the agricultural landscape. Although monthly sampling within 10-day windows may not capture short-term weather-driven fluctuations in stink bug activity, this approach effectively identified the major seasonal phenological patterns and parasitoid activity periods. *Halyomorpha halys* demonstrated a concentrated activity period in late summer, with increases in eggs and adults in August and September, a pattern similarly observed in other European regions, where late-season reproductive activity is influenced by the presence of two overlapping generations [46], temperature and photoperiod conditions [47,48,49]. The substantial increase in parasitism rates during September suggests a temporal synchronization between host and parasitoid populations, as also reported in Moraglio et al. [49] and Zapponi et al. [50], where both *T. japonicus* and *A. bifasciatus* populations follow the peak of *H. halys* eggs. This late-season peak in both reproduction and parasitism represents a critical period for stink bug population regulation as high parasitism potentially reduces the number of individuals entering diapause [51]. Additionally, successful parasitism during this period could enhance the overwintering parasitoid population, ensuring higher initial densities of natural enemies at the beginning of the following growing season. In contrast, *P. prasina* exhibited an earlier phenological pattern, a trend similarly observed in Northwest Italy [52], where peak egg laying in late spring coincides with high parasitism rates (>45%). This synchronization suggests a well-established host–parasitoid relationship, potentially indicative of long-term coevolution. Finally, *P. rufipes* showed a completely different cycle, overwintering as a second-instar nymph—a well-documented strategy in temperate European regions [53,54], where it shelters on the bark of tree trunks and branches [55]. The intense parasitism pressure during its reproductive period (>50%) was in line with findings from other studies [20,52], in which several parasitoid species, including *T. cultratus* and *A. sinicus*, emerged from the stink bug eggs.

Host plant utilization patterns indicated resource partitioning among the three stink bug species, with shared hosts but also distinct preferences. Throughout the life stages, the dominance of Sapindaceae and Rosaceae across the three insect species points to possible shared ecological niches, as observed in Berteloot et al. [7] for *H. halys* and *P. rufipes* adults and in Moraglio et al. [49] for egg masses of the three stink bug species. In this study, *H. halys* exhibited the broadest host range, demonstrating positive associations with Lamiaceae and Simaroubaceae, as reported in previous surveys in Europe [7,56] and North America [57]. None of the three species was found on gymnosperms, although plants of this taxon were considered during visual sampling. Previous studies confirmed that *H. halys* avoids certain plant taxa, particularly some gymnosperms, probably due to secondary metabolites or unsuitable structural features, while preferring nutritionally rich and architecturally complex hosts such as deciduous trees, shrubs, and crop plants [15,58]. On the other hand, *P. rufipes* was occasionally found on *Abies alba* Mill. trees during surveys in a forest reserve in Slovenia [59]. However, this stink bug species is also primarily associated with deciduous trees such as oak, alder, beech, hazel, and various species from the Rosaceae family [60,61,62]. Since *P. rufipes* is more commonly found in woodland and woodland edges, the significant difference in plant utilization between *H. halys* and *P. rufipes* observed in our study reflects inherent niche differences between these species, with *P. rufipes* showing a stronger association with forest habitats regardless of *H. halys* presence. In addition, this difference likely influences the distribution of their associated parasitoids, potentially reducing interspecific competition among parasitoid species and allowing for complementary parasitism across the different habitats. The strong affinity of all species for Sapindaceae, particularly *Acer* species, highlights the crucial role of this plant family in supporting pentatomid populations by providing critical resources for both feeding and oviposition, possibly due to their nutrient composition or canopy structure [7,15,56,57,63]. The distinct distribution patterns of adults, nymphs, and egg masses highlight that host plant use is not uniform across life stages but instead reflects stage-specific ecological requirements and behaviors. While adult stink bugs may exploit a broad range of host plants for feeding or mating, the concentration of egg masses on Sapindaceae underscores the reproductive importance of this plant family. This pattern is consistent with findings by Formella et al. [64], who observed that *H. halys* oviposition was strongly associated with particular tree species, even in the absence of adult aggregations. This suggests that oviposition site selection may be driven by specific cues or plant characteristics, independent of general host plant use. The broad distribution of both adults and nymphs across plant families emphasizes the role of landscape heterogeneity in supporting complete life cycles [65].

The results of the CCA indicate that the parasitoid community structure is shaped by both host availability and spatial–temporal factors. The elevational gradient in our study encompassed distinct environmental zone with elevation serving as a proxy for integrated climatic conditions including temperature regimes, precipitation patterns, and growing season duration that influence both host plant phenology and insect life cycle [66]. Strong ecological associations were observed between specific host–parasitoid pairs, such as *T. japonicus* with *H. halys* and *T. cultratus* with *P. rufipes*, consistent with findings from previous studies [49,50]. Spatial and temporal gradients emerged as significant determinants of community distribution, with elevation notably influencing species assemblages. Specifically, *P. rufipes*, *P. prasina*, and their primary parasitoids were positively associated with higher elevations compared to *H. halys*. Previous studies confirmed the presence of these species at higher altitudes than *H. halys* [20] and reported *P. rufipes* and *P. prasina* at elevations up to 1700 m and 1400 m, respectively [67,68,69]. Moreover, the CCA revealed a strong association between *H. halys* and *T. japonicus*, with both species positively correlated with smaller distance ranges from *T. japonicus* release sites selected within the nationwide classical biocontrol program. These findings validate the appropriate selection of those release sites as the correspondence between high *H. halys* densities and increased parasitoid presence demonstrates that sites were chosen to maximize establishment potential and parasitism effectiveness. This aligns with research demonstrating that the successful establishment of *T. japonicus* depends on host density thresholds and habitat stability [70,71]. Previous research conducted in this region [20] showed that both *H. halys* and *T. japonicus* were more frequently found at lower altitudes in landscapes dominated by perennial crops with limited semi-natural habitats while non-target hosts, such as *P. prasina*, were more common at higher elevations with greater semi-natural habitat availability. This suggests that landscape composition and elevation act as ecological filters. Similar to its main host, *T. japonicus* was more prevalent in orchards than in forests, mirroring patterns observed both in its native range [70] and in other invaded areas [72].

The constant activity of *A. bifasciatus* throughout the season suggests its potential role in regulating stink bug populations, particularly *H. halys* and *P. prasina*, as observed in Switzerland [50,73] and Italy [49,50,56]. However, as attested by their extended seasonal presence, *Trissolcus* spp. appear to constitute a well-functioning regulatory system capable of maintaining host population balance. Biological control efficiency could be affected by the activity of the hyperparasitoid *A. sinicus* increasing later in the season, resulting in potential impacts on primary parasitoid populations. Previous research reported that *A. sinicus* may reduce the effectiveness of *Trissolcus* spp. in controlling *H. halys* populations [22,74]. Notably, our findings suggest that *A. sinicus* may preferentially target *P. rufipes* eggs, which are more abundant in late season, particularly on Sapindaceae. This plant-specific pattern indicates that host plant selection may influence trophic interactions, potentially creating refuges or hotspots for hyperparasitism that could impact biological control dynamics across the agroecosystems [75,76].

The ecological interactions revealed in this study might have implications for integrated pest management strategies in agricultural and forest landscapes. The strong association of all three pentatomid species with Sapindaceae, particularly *Acer* species, aligns with findings from previous studies suggesting that these trees could serve as sentinel plants for stink bug population monitoring [49,50,52,56,77]. However, distinct temporal niches necessitate species-specific monitoring programs, with *H. halys* requiring attention in late summer and *P. prasina* in early season, as similarly observed in other studies [20,47,78]. The comparable stink bug assemblages observed between organic and IPM orchards suggest that management intensity may have limited influence on stink bug colonization patterns. This could be due to factors such as the low attractiveness of apple trees to stink bugs, rather than the efficacy of insecticide treatments, which were infrequent across all orchards.

The high parasitism rates, which reached up to 62.9% in *P. prasina* and remained substantial in all species, support the effectiveness of conservation biological control. Previous studies have indicated that synchronizing parasitoid activity with host reproduction optimizes natural enemy efficacy, reinforcing the importance of habitat management strategies that sustain parasitoid populations throughout critical host reproductive periods [79,80,81]. The influence of environmental gradients on host–parasitoid distributions, as demonstrated by the CCA, underlines the necessity of landscape-level management. Diversifying plant communities in managed ecosystems may help reduce overall pest pressure [82] while simultaneously supporting natural enemy populations [83,84]. Incorporating a range of host plant species that vary in their attractiveness to different stink bug species could disrupt pest colonization and reproduction while providing essential resources for natural enemies [85].

Our findings highlight the relationships within the agroecosystem, including host plants, stink bug life cycles, and parasitoid community dynamics. The observed patterns provide important baseline information for understanding which species are most relevant for pest management in this region and their temporal occurrence patterns. The strong associations between specific stink bug species and particular host plants, combined with the corresponding parasitoid communities, offer insights for targeted monitoring and intervention strategies during critical crop damage periods. These plants could function as natural enemy reservoirs, potentially enhancing the provision of biological control services in managed landscapes. Conserving these “hot spots” of natural enemy activity could strengthen the ecosystem resilience in integrated pest management programs. Further research is needed to understand the specific mechanisms driving host plant selection and the factors influencing parasitoid success on different host plants.

## 5. Conclusions

Our study highlighted the relationship between stink bugs, host plants, and egg parasitoids, demonstrating how plant composition and environmental features shape these interactions. The observed host plant use suggests resource allocation, with maple trees playing a central role in stink bug reproduction. Parasitism patterns indicate that biological control agents like *T. japonicus*, *T. cultratus*, and *Telenomus* spp. are strongly linked to host availability and environmental factors. Understanding these relationships can direct habitat management strategies to enhance parasitoid effectiveness and support sustainable pest control. Future work should focus on optimizing habitat conditions to improve the efficacy of natural enemies and reduce stink bug populations in agroecosystems.

## Figures and Tables

**Table 1:** Number of eggs, nymphs, and adults of *Halyomorpha halys*, *Palomena prasina,* and *Pentatoma rufipes* and percentage of parasitized eggs collected during two-year surveys.

		April	May	June	July	August	September
*H. halys*	Eggs (% parasitized)	0	0	249 (22.1)	654 (22.3)	962 (14.3)	1226 (44.2)
	Nymphs	0	0	72	69	65	82
	Adults	2	6	18	11	41	42
*P. prasina*	Eggs (% parasitized)	0	28 (0)	634 (62.9)	358 (44.1)	137 (0)	18 (0)
	Nymphs	0	0	13	47	26	7
	Adults	11	20	4	8	23	14
*P. rufipes*	Eggs (% parasitized)	0	0	56 (0)	16 (0)	14 (100)	556 (52)
	Nymphs	4	21	50	6	0	1
	Adults	0	0	15	91	97	28

**Table 2:** Standardized residuals from Chi-square test of independence between plant family and stink bug species. Positive and negative values indicate higher and lower than expected frequencies, respectively. Significant residuals (|residual| > 1.96) are marked with asterisks.

Plant Family	*H. halys*	*P. prasina*	*P. rufipes*
Adoxaceae	−0.74	0.34	0.65
Betulaceae	−1.20	0.72	0.86
Cornaceae	1.11	0.51	−2.06 *
Fabaceae	0.95	0.19	−1.50
Fagaceae	−2.15 *	0.08	2.85 **
Lamiaceae	2.49 *	−1.69	−1.58
Malvaceae	0.57	−0.72	0.00
Oleaceae	0.03	0.86	−0.97
Rosaceae	−0.20	0.64	−0.41
Sapindaceae	−0.58	−0.51	1.34
Simaroubaceae	2.23 *	−1.51	−1.41
Ulmaceae	−0.74	−0.63	1.68

* *p* < 0.05, ** *p* < 0.01.

**Table 3:** Pairwise comparison results (Bonferroni-adjusted alpha level (0.05/3) = 0.0167).

Comparison	Chi-Square *p*-Value	Fisher’s Exact *p*-Value	Significant After Bonferroni Correction
*P. rufipes* vs. *P. prasina*	0.0675	0.0650	No
*P. rufipes* vs. *H. halys*	2.77 × 10^−5^	0.0001	Yes
*P. prasina* vs. *H. halys*	0.0412	0.0272	No

## Data Availability

The original contributions presented in this study have been included in the article/Supplementary Materials. Further inquiries can be directed to the corresponding authors.

## Supplementary Materials

The following supporting information can be downloaded at: https://www.mdpi.com/article/10.3390/insects16070731/s1, Table S1: Survey sites’ geographical coordinates, altitudes, and distance ranges from *T. japonicus* release sites; Table S2: Stink bug presence on single host plants; Figure S1: Host–parasitoid network interactions.
