# Supplementary material for: Temporal Dynamics of Host Plant Use and Parasitism of Three Stink Bug Species: A Multi-Trophic Perspective
Source: Insects. 2025 Jul 17;16(7):731. doi: 10.3390/insects16070731 (PMC12295161; doi:10.3390/insects16070731)
Supplement: Supplementary file 1 [file insects-16-00731-s001.zip › insects-3686391-supplementary.pdf]

Table S1: Survey sites' geographical coordinates, altitudes and distance ranges from *T. japonicus* release sites

Site	Geographic coordinates (N, E)	Altitude (m a.s.l.)	Distance range from *T. japonicus* release site
**1**	46.385021, 11.286395	300	1000 – 5000 m
**2**	46.516366, 11.224336	360	300 – 1000 m
**3**	46.51681, 11.35365	332	300 – 1000 m
**4**	46.679801, 11.064966	640	300 – 1000 m
**5**	46.720068, 11.669110	667	1000 – 5000 m
**6**	46.635846, 11.199046	700	300 – 1000 m
**7**	46.548774, 11.492954	930	>5000 m
**8**	46.651643, 10.572912	893	>5000 m
**9**	46.805194, 11.952220	915	>5000 m
**10**	46.362128, 11.298278	222	300 – 1000 m
**11**	46.469982, 11.337642	243	1000 – 5000 m
**12**	46.655769, 11.149482	290	1000 – 5000 m
**13**	46.672551, 11.060623	514	1000 – 5000 m
**14**	46.700657, 11.649823	546	300 – 1000 m
**15**	46.796976, 11.669601	750	>5000 m
**16**	46.554720, 11.491648	970	>5000 m
**17**	46.794837, 11.928104	816	>5000 m
**18**	46.667307, 10.587580	935	>5000 m
**19**	46.383012, 11.288873	223	300 – 1000 m
**20**	46.646230, 11.183118	284	300 – 1000 m
**21**	46.344485, 11.278473	219	1000 – 5000 m
**22**	46.637905, 11.195173	700	1000 – 5000 m
**23**	46.664535, 11.194613	605	1000 – 5000 m
**24**	46.719069, 11.670230	655	1000 – 5000 m
**25**	46.542924, 11.494246	802	>5000 m
**26**	46.803347, 11.959907	932	>5000 m
**27**	46.659048, 10.579225	902	>5000 m

Table S2: Stink bug presence on single host plants

Host plant	Plant family	*H. halys*	*P. rufipes*	*P. prasina*
Acer campestre	Sapindaceae	0	1	0
Acer negundo		17	21	3
Acer platanoides		9	56	2
Acer pseudoplatanus		55	90	10
Koelreuteria paniculata		0	1	0
Ailanthus altissima	Simaroubaceae	10	0	1
Astragalus glycyphyllos	Fabaceae	0	0	3
Cercis siliquastrum		4	0	0
Lathyrus pratensis		0	0	2
Lotus corniculatus		1	0	0
Robinia pseudoacacia		11	1	3
Trifolium pratense		0	0	2
Betula pendula	Betulaceae	0	5	1
Betula pubescens		1	0	1
Corylus avellana		1	35	10
Carpinus betulus		0	0	1
Alnus glutinosa		0	1	2

Ostrya carpinifolia		32	1	1
Castanea sativa	Fagaceae	0	3	0
Fagus sylvatica		1	7	3
Quercus pubescens		0	5	2
Clerodendrum trichotomum	Lamiaceae	25	0	0
Galeopsis tetrahit		0	0	1
Cornus mas	Cornaceae	1	0	3
Cornus sanguinea		57	0	2
Hibiscus syriacus	Malvaceae	2	0	0
Tilia cordata		5	2	8
Tilia x europaea		3	2	0
Fraxinus excelsior	Oleaceae	30	7	5
Fraxinus ornus		20	7	5
Forsythia viridissima		1	0	2
Ligustrum lucidum		2	0	0
Ligustrum vulgare		4	0	5
Syringa vulgaris		3	6	20
Crataegus monogyna	Rosaceae	0	0	1
Geum urbanum		1	0	1
Malus baccata		1	0	0
Malus domestica		30	1	12
Prunus avium		37	20	4
Prunus cerasifera		0	1	1
Prunus cerasus		1	4	0
Prunus domestica		1	2	0
Prunus mahaleb		0	0	1
Prunus padus		7	0	3
Rosa canina		0	0	2
Rubus idaeus		0	0	6
Rubus sp.		2	0	1
Rubus ulmifolius		0	3	0
Sorbus aria		1	1	0
Sorbus aucuparia		0	1	1
Spiraea japonica		1	0	0
Ulmus glabra	Ulmaceae	2	9	0
Ulmus minor		1	0	0
Ulmus pumila		4	1	3
Viburnum lantana	Adoxaceae	0	11	2
Viburnum opulus		1	1	0
Viburnum tinus		1	0	0
Sambucus nigra		1	0	4
Clematis alpina	Ranunculaceae	1	0	0
Clematis vitalba		0	0	5
Populus alba	Salicaceae	0	1	3
Salix alba		1	0	1
Frangula alnus	Ramnaceae	1	0	2
Rhamnus cathartica		3	0	0
Brachypodium sylvaticum	Poaceae	0	0	1
Dactylis glomerata		0	0	1
Lolium perenne		0	0	4
Lapsana communis	Asteraceae	0	0	2
Taraxacum officinale		0	1	1
Celtis australis	Cannabaceae	1	0	0

Humulus lupulus		1	0	0
Hedera helix	Araliaceae	1	0	3
Hypericum calycinum	Clusiaceae	0	0	1
Juglans regia	Juglandaceae	0	3	0
Chenopodium album	Amarantaceae	2	0	0
Berberis vulgaris	Berberidaceae	0	0	2
Geranium robertianum	Geraniaceae	0	0	2
Fallopia convolvulus	Polygonaceae	0	0	2
Parthenocissus quinquefolia	Vitaceae	4	1	0
Philadelphus coronarius	Hydrangeaceae	0	0	1
Platanus x hispanica	Platanaceae	2	1	0
Euonymus europaeus	Celastraceae	3	0	0
Ribes rubrum	Grossulariaceae	1	0	0
Solanum nigrum	Solanaceae	0	0	1
Urtica dioica	Urticaceae	2	0	2

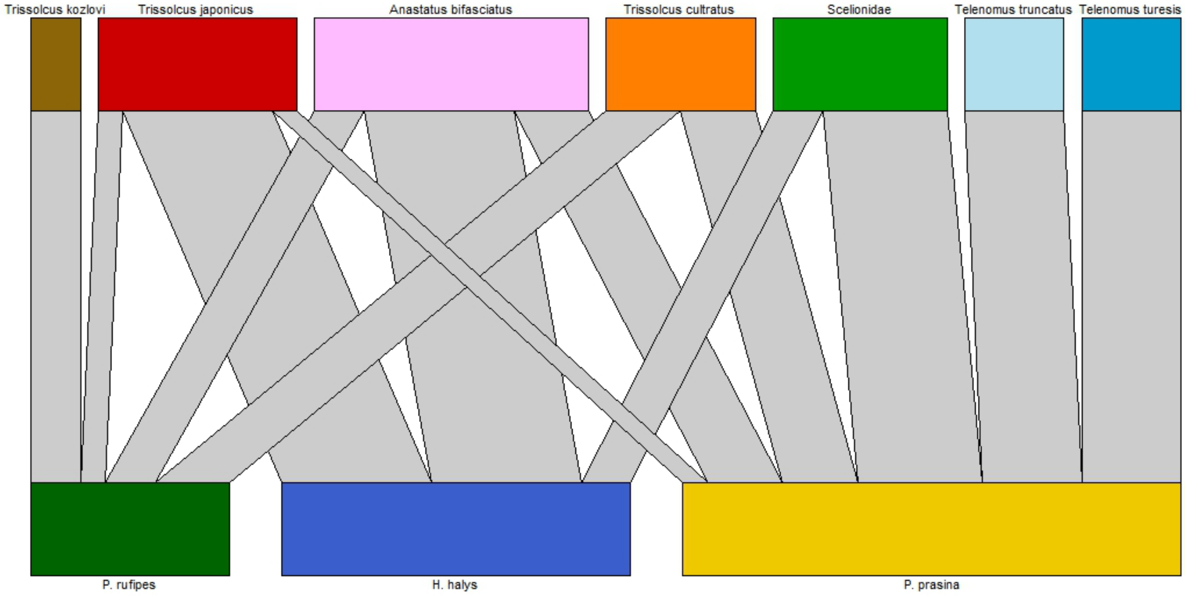

Figure S1: Host-parasitoid network interactions
